# Changes in Cyclin D1, cdk4, and Their Associated Molecules in Ischemic Pyramidal Neurons in Gerbil Hippocampus after Transient Ischemia and Neuroprotective Effects of Ischemic Preconditioning by Keeping the Molecules in the Ischemic Neurons

**DOI:** 10.3390/biology10080719

**Published:** 2021-07-28

**Authors:** Tae-Kyeong Lee, Dae Won Kim, Jae-Chul Lee, Cheol Woo Park, Hyejin Sim, Ji Hyeon Ahn, Joon Ha Park, Myoung Cheol Shin, Jun Hwi Cho, Choong-Hyun Lee, Moo-Ho Won, Soo Young Choi

**Affiliations:** 1 Department of Biomedical Science and Research Institute of Bioscience and Biotechnology, Hallym University, Chuncheon 24252, Korea; tk_lee@hallym.ac.kr; 2 Department of Biochemistry and Molecular Biology and Research Institute of Oral Sciences, College of Dentistry, Kangnung-Wonju National University, Gangneung 25457, Korea; kimdw@gwnu.ac.kr; 3 Department of Neurobiology, School of Medicine, Kangwon National University, Chuncheon 24341, Korea; anajclee@kangwon.ac.kr (J.-C.L.); flfhflfh@naver.com (C.W.P.); janny20@naver.com (H.S.); jh-ahn@ysu.ac.kr (J.H.A.); 4 Department of Physical Therapy, College of Health Science, Youngsan University, Yangsan 50510, Korea; 5 Department of Anatomy, College of Korean Medicine, Dongguk University, Gyeongju 38066, Korea; jh-park@dongguk.ac.kr; 6 Department of Emergency Medicine, Kangwon National University Hospital, School of Medicine, Kangwon National University, Chuncheon 24289, Korea; dr10126@naver.com (M.C.S.); cjhemd@kangwon.ac.kr (J.H.C.); 7 Department of Pharmacy, College of Pharmacy, Dankook University, Cheonan 31116, Korea; anaphy@dankook.ac.kr

**Keywords:** ischemic preconditioning, cyclin D1/cdk4 signals, transient global ischemia, hippocampus, pyramidal cells, gerbil

## Abstract

**Simple Summary:**

Cyclin D1 and cyclin-dependent kinase 4 (cdk4) is implicated in neuronal death induced by various pathological conditions. Ischemic preconditioning (IPC) confers neuroprotective effect, but underlying mechanisms have been poorly addressed. In this study, IPC protected pyramidal neurons (cells) in gerbil hippocampus after transient ischemia. Additionally, IPC controlled expressions of cyclin D1, cdk4, phosphorylated retinoblastoma (*p*-Rb), and E2 promoter binding factor 1 (E2F1). In particular, the expression of p16INK4a was not different by IPC. These findings indicate that cyclin D1/cdk4-related signals may play important roles in events in neurons related to damage/death following ischemic insults. Especially, the preservation of p16INK4a by IPC may be crucial in attenuating neuronal death/damage or protecting neurons after brain ischemic insults.

**Abstract:**

Inadequate activation of cell cycle proteins including cyclin D1 and cdk4 is involved in neuronal cell death induced by diverse pathological stresses, including transient global brain ischemia. The neuroprotective effect of ischemic preconditioning is well-established, but the underlying mechanism is still unknown. In this study, we examined changes in cyclin D1, cdk4, and related molecules in cells or neurons located in Cornu Ammonis 1 (CA1) of gerbil hippocampus after transient ischemia for 5 min (ischemia and reperfusion) and investigated the effects of IPC on these molecules after ischemia. Four groups were used in this study as follows: sham group, ischemia group, IPC plus (+) sham group, and IPC+ischemia group. IPC was developed by inducing 2-min ischemia at 24 h before 5-min ischemia (real ischemia). Most pyramidal cells located in CA1 of the ischemia group died five days after ischemia. CA1 pyramidal cells in the IPC+ischemia group were protected. In the ischemia group, the expressions of cyclin D1, cdk4, phosphorylated retinoblastoma (*p*-Rb), and E2F1 (a transcription factor regulated by *p*-Rb) were significantly altered in the pyramidal cells with time after ischemia; in the IPC+ischemia group, they were controlled at the level shown in the sham group. In particular, the expression of p16^INK4a^ (an endogenous cdk inhibitor) in the ischemia group was reversely altered in the pyramidal cells; in the IPC+TI group, the expression of p16^INK4a^ was not different from that shown in the sham group. Our current results indicate that cyclin D1/cdk4-related signals may have important roles in events in neurons related to damage/death following ischemia and reperfusion. In particular, the preservation of p16^INK4a^ by IPC may be crucial in attenuating neuronal death/damage or protecting neurons after brain ischemic insults.

## 1. Introduction

Delayed neuronal cell death in the brain is an important component of ischemic damage following transient global brain ischemia, and this death occurs well in pyramidal cells located in CA1 among all subfields (CA1-3) in the hippocampus at a few days after the ischemia, showing that pyramidal neuronal cells in CA3 are less affected [1,2]. The major pattern of delayed neuronal cell death in CA1 after transient ischemia, which occurs at 4–5 days after transient ischemia, is apoptosis rather than necrosis in terms of morphology and biochemistry [3,4,5]. However, studies have demonstrated that delayed neuronal death progresses by displaying elements of both necrosis- and apoptosis-like characteristics [6,7].

Ischemic preconditioning (IPC) refers to the ability of brief ischemic period and can mobilize protective mechanisms against subsequent morbid ischemia-reperfusion injury [8,9,10,11,12,13]. Brains have endogenous mechanisms to be able to defend themselves against ischemic insults [14,15]. These innate pathways in the brains are able to be activated by IPC, namely, exposure to sub-lethal stressor (IPC) triggers alterations in the expressions of genes and proteins that lead to tolerance to subsequent lethal ischemia-reperfusion injury that usually kills vulnerable neurons [16,17]. This phenomenon in ischemic brains is termed ‘ischemic tolerance’ [18]. Ischemic tolerance is a primary target in strategies for neuroprotection against ischemic injury, its molecular mechanisms of ischemic tolerance have not fully been reported yet [19].

Cyclin D1 is a crucial regulator of the cell cycle, in particular through G1/S phase, by stimulation of the activity of cdk4, which phosphorylates retinoblastoma (Rb) protein [20]. Consequently, Rb protein dissociates from E2 promoter binding factor 1 (E2F1), and this facilitates gene transcription needed for S-phase progression [21]. Generally, neuronal cells do not undergo cell proliferation and are usually downregulated in cell cycle proteins [22]. In ischemic brains, neuronal cells are triggered for cell cycle reentry after brain ischemia [23], and the aberrant activation of the cell cycle can be induced to enter apoptosis in neuronal cells following ischemia-reperfusion, because the neurons are terminally differentiated cells that are not able to undergo proliferation [24,25]. Cyclin D1 expression is increased in response to diverse neurotoxic agents, which is implicated in neuronal cell death [26,27]. In animal models of brain and spinal cord ischemia, cyclin D1 and cdk4 expressions are increased in neurons and/or glial cells after focal brain ischemia in mice and rats [28], global brain ischemia in rats [29], and spinal cord ischemia in rabbits [30]. However, conflicting results for the role of cyclin D1 have persisted. For example, enhanced expression of cyclin D1 in the hippocampus induced by transient global brain ischemia in rats is localized to apoptotic granule cells located in the dentate gyrus, but not CA1 cells [31]. On the other hand, other studies have reported that increased cyclin D1 expression is preferentially enhanced in the vulnerable CA1 of global ischemia-induced rats, and this cyclin D1 induction occurs in neurons before the appearance of chromosomal DNA fragmentation [29]. Anyway, the upregulation of cyclin D1 may occur in dying neurons, showing that cyclin D1-related signal is critically involved in neuronal death following ischemic injury.

The degree and/or pattern of neuronal death/loss following ischemic insults is dependent on various factors including types of ischemia in brains (permanent focal ischemia, transient focal ischemia, transient global ischemia, transient forebrain ischemia, etc.), ischemic duration, animal species, etc.). Transient focal brain ischemia develops infarct lesion in the striatum and neocortex [32,33]; whereas transient ischemia in the forebrain evokes selective neuronal cell death in the hippocampal CA1 and neocortex [34,35]. In this regard, different approaches to studying neuroprotective mechanisms should be applied.

Until now, many explanations of IPC-mediated neuroprotective effects against ischemic insults have been suggested. First of all, IPC-mediated neuroprotection in ischemic brains may include the regulation of cyclin D1 and its downstream proteins. However, we need to fully address whether IPC-mediated cyclin D1 expression contributes to neuroprotective effects against injury induced by ischemia and reperfusion. Thus, we examined changes in transient ischemia-induced expressions of cell cycle proteins including cyclin D1, cdk4, p16^INK4a^ (the endogenous cdk inhibitor), *p*-Rb and E2F1, and investigated IPC-mediated neuroprotective effect against subsequent transient ischemic insults to establish the crosstalk between them.

## 2. Materials and Methods

### 2.1. Experimental Animals

In this experiment, male gerbils because estrogen protects neuronal loss in the hippocampus following ischemic insult in gerbils [36]. A total of 96 male gerbils (6-month-old; body weight, 64–76 g) bred in the Experimental Animal Center of Kangwon National University (Chuncheon, Korea) were used. They had been bred in pathogen-free condition with adequate temperature (23 °C) and humidity (60%). The protocol of this experiment was approved (approval number, KW-151127–1) by the Institutional Animal Care and Use Committee (IACUC). The content of this protocol kept the guidelines described in the “Current International Laws and Policies” in the “Guide for the Care and Use of Laboratory Animals” of The National Academies Press (8th Ed., 2011).

### 2.2. Experimental Groups

Four groups were used: (1) sham transient ischemia (TI) operated group (sham group; *n* = 12) did not receive TI; (2) TI operated group (TI group; *n* = 36) was given a 5-min TI; (3) IPC treated and sham TI operated group (IPC+sham group; *n* = 12) received IPC (a 2-min TI before a 5-min TI), and was given no TI; and (4) IPC+TI group (*n* = 36) was subjected to a 5-min TI following IPC.

### 2.3. Surgery of IPC and TI

TI was advanced in the forebrain according to our published procedure [17]. The anesthesia of the gerbils was induced with 2.5% isoflurane (in 33% oxygen and 67% nitrous oxide). After the confirmation of the anesthesia, left and right common carotid arteries that give blood to the brains- were searched out from the carotid sheath, and they were ligated by using clips. To confirm the complete stop of blood flow, the central arteries located in the retinae were monitored by using an ophthalmoscope. A 2-min and a 5-min occlusion was performed for IPC and TI, respectively. Body (rectal) temperature was measured with temperature probe (TR-100) from Fine Science Tools (Foster City, CA, USA) and controlled at normothermic condition (37 ± 0.5 °C) by using thermometric blankets. Normothermia was kept before and during the TI surgery. When the gerbils were fully recovered from the anesthesia, they were kept in thermal incubators of Mirae Medical Industry (Seoul, Korea) until the gerbils were euthanized for the experiments. The gerbils with sham TI operation received the same TI surgery without the ligation of the arteries.

### 2.4. Western Blot

For Western blot, the gerbils (*n* = 5 at each time in each group) were sacrificed at 1 day, 2 days and 5 days after TI, because the pyramidal cells of CA1 die at 4 or 5 days after a 5-min TI. They were anesthetized for euthanasia with 200 mg/kg of pentobarbital sodium of JW Pharmaceutical (Seoul, Korea)

The cytosolic and nuclear fractions were obtained according to a published method [37] and Western blot analyses for cyclin D1, cdk4, p16^INK4a^ *p*-Rb, E2F1, in CA1 were done according to a method [38]. In short, the tissues of CA1 were homogenized, and their protein levels in the CA1 supernatants were determined with Micro BCA protein assay kit obtained from Pierce Biotechnology (Rockford, IL, USA). Namely, the membranes were incubated in rabbit anti-cyclin D1 (diluted 1:1000) (Santa Cruz Biotechnology Inc., Santa Cruz, CA, USA), rabbit anti-cdk4 (diluted 1:1000) (Cell Signaling Technology, Danvers, MA, USA), rabbit anti-p16^INK4a^ (diluted 1:100) (Santa Cruz Biotechnology Inc.), rabbit anti-Rb (phospho Ser780) (diluted 1:1000) (Cell Signaling Technology), and rabbit anti-E2F1 (diluted 1:1000) (Santa Cruz Biotechnology Inc), rabbit anti-lamin B (diluted 1:1500) (Santa Cruz Biotechnology Inc.), mouse anti-α-tubulin (diluted 1:1000) (Abcam, Cambridge, UK), and mouse anti-β-actin (diluted 1:2000) (Sigma-Aldrich, St. Louis, MO, USA). Finally, the bands were visualized by using ECL kit obtained from Pierce Biotechnology (Waltham, MA, USA).

The analysis of the results of Western blot was carried out as described previously [39]. Briefly, the blots were scanned, and the densitometric analyses were done to evaluate the bands with Scion Image software of Scion Corp (Frederick, MD, USA). In this experiment, the rate of each target protein expression was normalized through the corresponding expression rate of β-actin.

### 2.5. Tissue Preparation for Histological Study

For histopathological study, the gerbils (*n* = 7 at each time) in each group were sacrificed 1 day, 2 days and 5 days after TI, because CA1 pyramidal cells die at 4 or 5 days after a 5-min TI.

According to a method [39], in brief, the gerbils (*n* = 7 at each time in each group) were anesthetized with 200 mg/kg of pentobarbital sodium of JW Pharmaceutical (Seoul, Korea). After confirming the deep anesthesia, their brains were perfused transcardially to be fixed with 4% paraformaldehyde (in 0.1 M phosphate-buffer (PB), pH 7.4). Thereafter, the brain tissues containing the hippocampi were frontally cut into 30-μm thickness in cryostat (CM1900 UV) (Leica, Germany).

### 2.6. Histochemistry Using Cresyl Violet

In this experiment, Cresyl Violet (CV) histochemical staining was performed in order to examine the distribution and damage of cells located in gerbil hippocampus following sham and TI operation. In brief, aa described in our published paper [39]. One % (*w*/*v*), of CV acetate obtained from Sigma-Aldrich (St. Louis, MO, USA) was made, and glacial acetic acid (0.28%) was added to this CV solution. Thereafter, the prepared brain sections were stained with the CV solution, dehydrated and mounted with coverglasses.

Cellular damage in CA1 after TI was observed according to a published method [39]. In short, seven sections/gerbil were examined by using light microscope (AxioM1) of Carl Zeiss (Germany) with camera (Axiocam) of Carl Zeiss connected to PC.

### 2.7. Fluoro-Jade B Histofluorescence

Fluoro-Jade B (F-J B) has been used to detect cellular or neuronal degeneration/death after ischemic insults. In this study, F-J B histofluorescence was performed in CA1, as previously described [40]. The prepared brain sections were immersed in 1% sodium hydroxide (Junsei Chemical Co., Ltd., Tokyo, Japan) for 10 min and transferred to 0.06% potassium permanganate (Sigma-Aldrich, St. Louis, MO, USA) for 20 min. Thereafter, they were stained with 0.0004% F-J B (Histochem, Jefferson, AR, USA) for 40 min, and the sections were briefly rinsed and reacted on a heated (about 50 °C) slide warmer.

The quantitative analysis of neurons or cells positive to F-J B was done for neuronal loss/death. As previously describe [39], in brief, the numbers of F-J B positive cells were counted in a 200 µm^2^ of CA1 including the pyramidal layer by using epifluorescent microscope of Carl Zeiss (Göttingen, Germany) with blue excitation light (450–490 nm). Finally, the cell count was performed using image analyzing system (Optimas 6.5) of CyberMetrics (Scottsdale, AZ, USA).

### 2.8. Immunohistochemistry

In this study, immunohistochemistry was performed for neuronal nuclei (NeuN; a neuron-specific soluble nuclear antigen), cyclin D1, cdk4, p16^INK4a^, Rb and E2F1 in the hippocampal CA1 after TI according to a published procedure [39]. In short, the brain sections were incubated in each antibody for 9 h at 4 °C. Primary antibodies used in this study were mouse anti-NeuN (diluted 1:1000) (Chemicon International, Temecula, CA, USA), rabbit anti-cyclin D1 (diluted 1:100) (Santa Cruz Biotechnology Inc., Santa Cruz, CA, USA), rabbit anti-cdk4 (diluted 1:100) (Santa Cruz Biotechnology Inc.), rabbit anti-p16^INK4a^ (diluted 1:100) (Santa Cruz Biotechnology Inc.), rabbit anti-Rb (phospho Ser-780) (diluted 1:100) (Abcam, Cambridge, UK), and rabbit anti-E2F1 (diluted 1:100) (Santa Cruz Biotechnology Inc.). Thereafter, the sections were briefly rinsed, incubated in secondary antibodies (Vector Laboratories Inc, Burlingame, CA, USA) and developed by using Vectastain ABC (Vector Laboratories Inc, Burlingame, CA, USA). Finally, the immunoreacted hippocampal sections were visualized by using 3,3′-diaminobenzidine solution (DAB).

The count of NeuN immunoreactive cells (neurons) was done according to a published method [39]. Namely, the digital images of NeuN immunoreactive cells were captured from 7 sections/gerbil by using light microscope of AxioM1 (Carl Zeiss, Germany) with digital camera of Axiocam (Carl Zeiss). The count of these cells was done like the method described in Section 2.7.

The analyses of cyclin D1, cdk4, p16^INK4a^, Rb and E2F1 immunoreactivity were carried out according to a method [39]. Namely, the image of each immunoreactive structure was captured like the above-described method. For the evaluation of the immunoreactivity, optical density (OD) was obtained after converting the color image into mean gray level using a formula − OD = log (256/mean gray level). The brightness and contrast of each immunoreactivity was evaluated as percent (relative optical density, ROD) by using Adobe Photoshop version 8.0 of San Jose (CA, USA) and analyzed by using NIH Image J software (Bethesda, MD, USA).

### 2.9. Double Immunofluorescence

In this experiment, cdk4 immunoreactivity was newly shown in CA1 cells at 5 days after TI. To confirm cell type of these cells, the sections obtained at 5 days post-TI were processed by double immunofluorescence using rabbit anti-cdk4 (diluted 1:50) (Santa Cruz Biotechnology), mouse anti-glial fibrillary acidic protein (GFAP) (diluted 1:100) (Chemicon International, Temecula, CA, USA) for astrocytes or mouse anti-ionized calcium-binding adapter molecule 1 (Iba-1) (diluted 1:110) (Wako, Osaka, Japan) for microglia by using the method described in Section 2.8. After washing them, the sections were incubated in the mixture of FITC-conjugated goat anti-rabbit IgG (diluted 1:200) (Jackson ImmunoResearch, West Grove, PA, USA) and Cy3-conjugated goat anti-mouse IgG (diluted 1:200) (Jackson ImmunoResearch) for 2 h at room temperature.

To identify the cell type of double immunoreactive cells, the stained sections were observed using confocal microscope (LSM510 META NLO) (Carl Zeiss, Germany).

### 2.10. Statistical Analysis

In this experiment, the sample size was at least 5 (for western study) and 7 (for histological study) rats per group with an alpha error of 0.05 and a power of >80%. The sample size was calculated with power calculator. All data are presented as mean ± standard deviation (SD). In addition, a multiple-sample comparison was applied for testing the differences between groups: ANOVA and Tukey multiple range test as post hoc test using the criterion of the least significant differences. *p* < 0.05 was considered as statistical significance.

## 3. Results

### 3.1. CA1 Pyramidal Cells Died after TI and IPC Protected the Cells from TI

#### 3.1.1. Finding by CV Histochemistry

**Figure 1:**
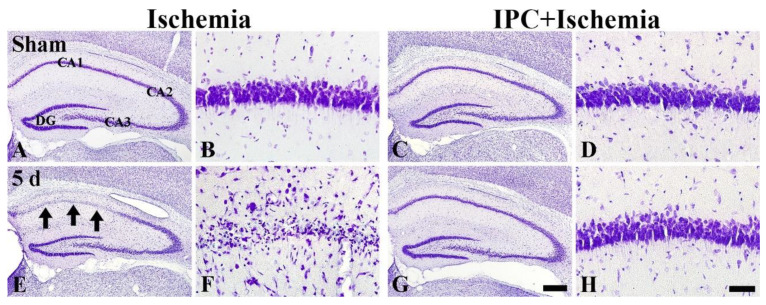
Cresyl Violet (CV) histochemistry in gerbil hippocampus of the sham (**A**,**B**), TI (**C**,**D**), IPC+sham (**E**,**F**), and IPC+TI (**G**,**H**) groups. Pyramidal cells are severely damaged (arrows in (**E**), and (**F**)) in CA1, not CA2/3, at 5 days after TI. On the other hand, CA1 pyramidal neurons in the IPC+TI group (**G**,**H**) are similar to those shown in the sham group. Scale bar = 400 µm (**A**,**C**,**E**,**G**), and 50 µm (**B**,**D**,**F**,**H**).

TI-induced cell damage and IPC-mediated protection was observed in gerbil hippocampal CA1 using CV histochemistry as shown in Figure 1. CV-positive (CV^+^) cells in the sham group were well defined in each layer of all subfields (CA1-3) (Figure 1A). In particular, the CV^+^ cells located in the pyramidal layer (stratum pyramidale), which are principal neurons and called pyramidal cells or neurons, were large in their size and pyramidal or round in their morphology (Figure 1B). After TI, the morphology of CV^+^ pyramidal cells located in CA1-3 were not altered until 3 days post-TI (data not shown). Five days after TI. However, CV^+^ pyramidal neurons were severely damaged in CA1, but CV^+^ pyramidal cells located in CA2/3 were intact (Figure 1E). The damaged CV^+^ CA1 pyramidal cells had hardly cytoplasm and contained pycnotic nucleus at high magnification of the ischemic CA1 (Figure 1F). In the IPC+sham group, CV^+^ pyramidal neurons located in CA1-3 were similar to those shown in the sham group (Figure 1C,D). In the IPC+TI group, CV^+^ pyramidal neurons were also not different from those found in the IPC+sham group after TI (Figure 1G,H). This finding indicates that IPC protects CA1 pyramidal neurons from TI.

#### 3.1.2. Findings by NeuN Immunohistochemistry and F-J B Histofluorescence

**Figure 2:**
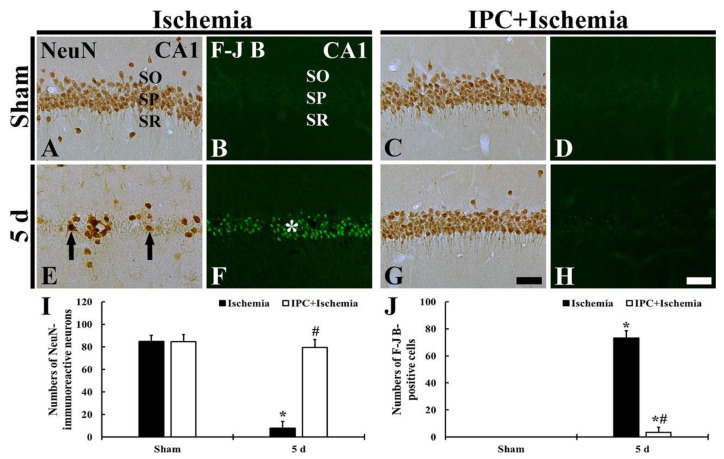
(**A**–**H**) Immunostaining of NeuN (**A**,**C**,**E**,**G**) and F-J B histofluorescence (**B**,**D**,**F**,**H**) in CA1 of the sham (**A**,**B**), TI (**C**,**D**), IPC+sham (**E**,**F**), and IPC+TI (**G**,**H**) groups at 5 days after TI. A few NeuN^+^ cells (arrows in (**E**)) are found in the stratum pyramidale (SP) of the TI group. In the IPC+TI group, however, the immunostaining of NeuN is similar to that in the sham. Many F-J B^+^ CA1 pyramidal cells (white asterisk in (**F**)) are detected at 5 days post-TI, however, F-J B^+^ cells are hardly shown. SO, stratum oriens; SR, stratum radiatum. Scale bar = 50 µm. (**I**,**J**) Mean numbers of NeuN^+^ (**I**) and F-J B^+^ (**J**) CA1 pyramidal neurons in the SP. The bars indicate the means ± SD (*n* = 7, * *p* < 0.05 versus sham; ^#^ *p* < 0.05 versus TI group).

TI-induced neuronal cell death (loss) and IPC-mediated protection in CA1 was observed using NeuN immunostaining and F-J B histofluorescence. As shown in Figure 2, pyramidal cells located in CA1 of the sham group were well immunopositive to NeuN, and, in this group, any F-J B^+^ cells were not detected in CA1(Figure 2A,B). However, in the TI group, NeuN^+^ CA1 pyramidal cells were dramatically decreased (about 9% of the sham) at 5 days after TI, and, at this time, numerous F-J B^+^ cells were shown in the stratum pyramidale (Figure 2E,F,I). In the IPC+sham group, immunostaining of NeuN in CA1 was similar to that in the sham group, showing that any F-J B^+^ CA1 pyramidal cells were not found (Figure 2C,D). In the IPC+TI group, most of NeuN^+^ CA1 pyramidal cells were protected by IPC (about 84% of the sham) at 5 days after TI, and, at this time, the numbers of F-J B^+^ CA1 pyramidal cells were markedly reduced (about 8% of the TI group) (Figure 2G,H,J).

### 3.2. TI Altered Cyclin D1 Expression and IPC Protected the Change in CA1 Pyramidal Cells

#### 3.2.1. Cyclin D1 Immunoreactivity

**Figure 3:**
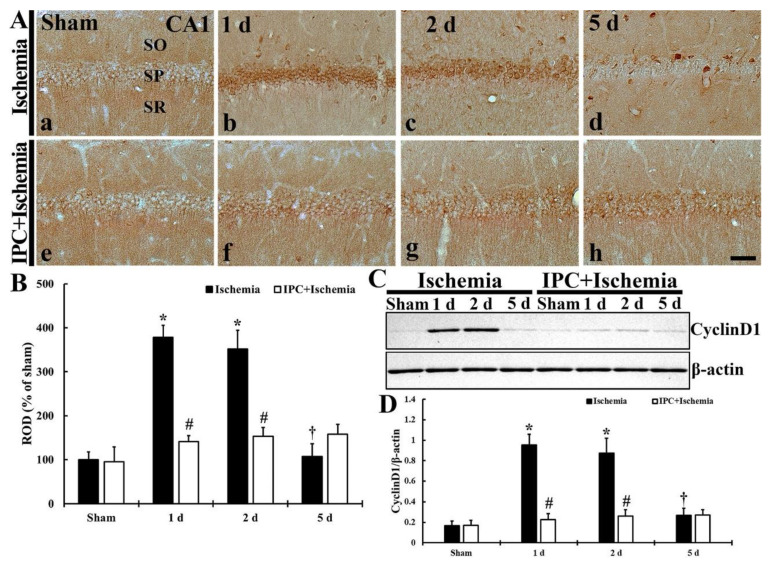
(**A**) Immunostaining of cyclin D1 in CA1 of the TI (upper column) and IPC+TI (lower column) groups at sham (a,e), 1 day (b,f), 2 days (c,g), and 5 days (d,h) after TI. Cyclin D1 immunoreactivity is increased in pyramidal cells located in the stratum pyramidale (SP) at 1 and 2 days after TI. In the IPC+TI group, cyclin D1 immunoreactivity is similar to that found in the sham group. SO, stratum oriens; SR, stratum radiatum. Scale bar = 50 μm. (**B**) Quantitative graph of cyclin D1 immunoreactivity in pyramidal cells. Relative optical density (ROD) was calibrated as percent with the sham (100%). The bars represent means ± SD (*n* = 7, * p < 0.05 versus sham group; *^#^ p* < 0.05 versus TI group; ^†^ *p* < 0.05 versus prior time point of each group). (**C**) Western blot of cyclin D1 (37 kDa) in CA1 of the sham, TI, IPC+sham and IPC+TI groups at 1, 2, and 5 days after TI. β-actin was used as a protein loading control. (**D**) Relative band intensity of total cyclin D1 level. Cyclin D1 level is significantly low in the IPC+TI group compared with the TI group. The bars represent means ± SD (*n* = 7, * *p* < 0.05 versus sham group; ^#^ *p* < 0.05 versus TI group; ^†^ *p* < 0.05 versus prior time point of each group).

TI-induced change and IPC-mediated protection of cyclin D1 immunoreactivity in CA1 was investigated, as shown in Figure 3A,B. Cyclin D1 immunoreactivity, in the sham group, was weak in pyramidal cells (Figure 3A(a)). In the TI group, cyclin D1 immunoreactivity was markedly increased (378.8% and 351.7% of the sham) in the pyramidal cells at 1 and 2 days after TI (Figure 3A(b,c),B), and, at 5 days after TI, cyclin D1 immunoreactivity in the pyramidal cells was weakened (107.2% of the sham) (Figure 3A(d),B). In the IPC+sham group, cyclin D1 immunoreactivity in the pyramidal neurons was similar to that found in the sham group. In the IPC+TI group, cyclin D1 immunoreactivity in the pyramidal neurons was not different from that found in the sham group until 5 days post- TI (Figure 3A(e–h),B).

#### 3.2.2. Cyclin D1 Protein Level

TI-induced alteration and IPC-mediated protection of cyclin D1 protein level in CA1 was investigated, as shown Figure 3C,D. The level of cyclin D1 protein was very weak in the sham group (Figure 3C). The level of cyclin D1 protein was upregulated (570.2% and 522.9% of the sham) at 1 and 2 days after TI. Five days after TI, the level was low (159.9% of the sham) (Figure 3C,D). In the IPC+sham group, cyclin D1 protein level was similar to that in the sham group (Figure 3C,D). Also, the level of cyclin D1 protein in the IPC+TI group was not changed at any times after TI when compared with that shown in the sham group (Figure 3C,D).

### 3.3. TI Altered cdk4 Expression and IPC Protected the Change in CA1 Pyramidal Cells

#### 3.3.1. Cdk4 Immunoreactivity

**Figure 4:**
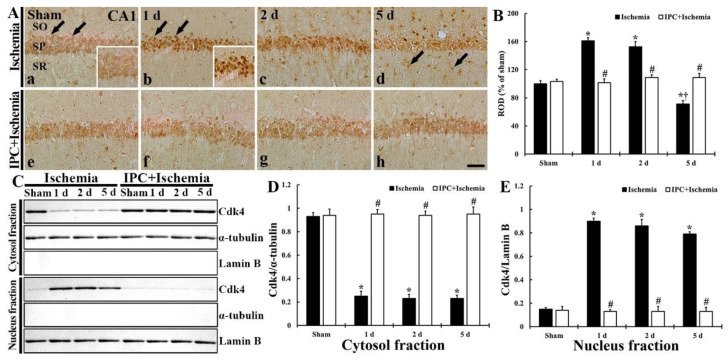
(**A**) Immunostaining of cdk4 in CA1 of the TI (upper column) and IPC+TI (lower column) groups at sham (a,e), 1 day (b,f), 2 days (c,g), and 5 days (d,h) after TI. Cdk4 immunoreactivity detected in the sham group is mainly found in the cytoplasm (arrows in A(a)) of the pyramidal cells. In the TI group, Cdk4 immunoreactivity in the pyramidal cells is translocated into their nuclei (arrows in A(b)) after TI, showing that, at 5 days, Cdk4 immunoreactivity is low in the pyramidal cells and newly shown in non-pyramidal cells (arrows in A(d)). In the IPC+sham and IPC+TI groups, cdk4 immunoreactivity in the pyramidal cells is similar to that shown in the sham group. Scale bar = 50 µm. (**B**) ROD graph of cdk4 immunoreactivity in CA1 pyramidal cells. ROD was calibrated as percent (sham group, 100%). The bars represent means ± SD (*n* = 7, * *p* < 0.05 versus sham; ^#^ *p* < 0.05 versus TI group; ^†^ *p* < 0.05 versus prior time point of each group). (**C**) Western blot of cdk4 (30 kDa) protein level in CA1 of the TI and IPC+TI groups at sham, 1 day, 2 days, and 5 days after TI. α-tubulin and lamin B are used for standardizing cytosol and nucleus protein loading. (**D**,**E**) Relative band intensity of cdk4 level in cytosol (**D**) and nucleus (**E**). The bars represent means ± SD (*n* = 7, * *p* < 0.05 versus sham; ^#^ *p* < 0.05 versus TI group).

Subsequently, the cellular distribution of cdk4 in ischemic CA1 pyramidal cells with or without IPC was examined, as shown in Figure 4A,B. In the sham group, weak cdk4 immunoreactivity was found in both cytoplasm and nuclei of the pyramidal cells (Figure 4A(a)). In the TI group, strong cdk4 immunoreactivity was found in the nuclei, not cytoplasm, of the pyramidal neurons at 1 and 2 days after TI (Figure 4A(b,c)), showing that, at these times, the cdk4 immunoreactivity was significantly increased (161.0% and 152.7% of the sham, respectively) (Figure 4B). Five days after TI, cdk4 immunoreactivity in the pyramidal cells was too weak (71.3% of the sham) (Figure 4A(d),B). In particular, at this time after TI, cdk4 immunoreactivity was newly expressed in non-pyramidal cells located in the stratum oriens and radiatum (Figure 4A(d)). In the IPC+sham group, cdk4 immunoreactivity of the pyramidal cells was similar to that found in the sham group (Figure 4A(e),B). In the IPC+TI group, cdk4 immunoreactivity shown in the pyramidal neurons did not change after TI when compared with that found in the IPC+sham group (Figure 4A(f–h),B).

#### 3.3.2. Cdk4 Protein Level

TI-induced alteration and IPC-mediated protection of cdk4 protein level in CA1 was examined, as shown Figure 4C–E. Cdk4 protein in CA1 of the sham group was contained in both cytosol and nucleus fractions (Figure 4C,D). In the TI group, cdk4 protein level was dramatically decreased (598.3% at 1 day, 573.3% at 2 days and 526.7% at 5 days of the sham), but significantly increased (26.9% at 1 day, 24.7% at 2 days and 24.7% at 5 days of the sham) in the nucleus fraction (Figure 4C–E). cdk4 protein level in the IPC+sham group, was similar to that found in the sham group, and cdk4 protein levels detected in the IPC+TI group was not altered after TI (Figure 4C,D).

#### 3.3.3. New Expression of cdk4 in Astrocytes

**Figure 5:**
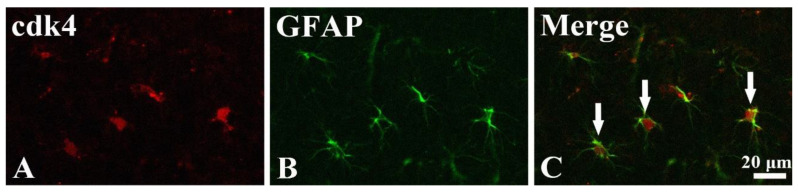
Double immunofluorescence for cdk4 ((**A**); red), GFAP ((**B**); blue), and merged image (**C**) in the stratum radiatum at 5 days after TI. Cdk4 immunofluorescence is co-localized with astrocytes (white arrows in (**C**)) which are immunostained with GFAP. Scale bar = 20 μm.

To determine the type of the non-pyramidal cells containing cdk4, which were found in the stratum oriens and radiatum at 5 days after TI, double-labeling study was done. As shown in Figure 5. cdk4 was co-localized with astrocytes which were immunostained with GFAP (Figure 5). In this study, cdk4 immunoreactivity was not found in microglia, which was identified by immunostaining for Iba-1 (data not shown).

### 3.4. TI Altered p16^INK4a^ Expression and IPC Protected the Change in CA1 Pyramidal Cells

#### 3.4.1. p16^INK4a^ Immunoreactivity

**Figure 6:**
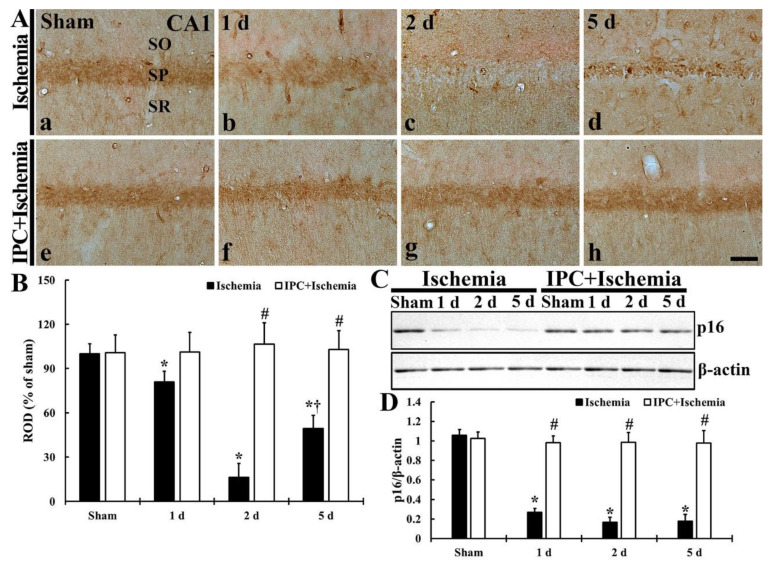
(**A**) Immunostaining of p16^INK4a^ in CA1 of the TI (upper column) and IPC+TI (lower column) groups at sham (a,e), 1 day (b,f), 2 days (c,g), and 5 days (d,h) after TI. p16^INK4a^ immunoreactivity in the pyramidal cells is gradually decreased after TI. In the IPC+TI group, however, p16^INK4a^ immunoreactivity in the pyramidal cells is well preserved after TI. Scale bar = 50 μm. (**B**) ROD graph of p16^INK4a^ immunoreactivity in pyramidal cells. ROD was calibrated as percent (sham group, 100%). The bars represent means ± SD (*n* = 7, * p < 0.05 versus sham; *^#^ p* < 0.05 versus TI group; ^†^ *p* < 0.05 versus prior time point of each group). (**C**) Western blot of p16^INK4a^ (16 kDa) in CA1 of the TI and IPC+TI group at sham, 1 day, 2 days and 5 days after TI. β-actin was used as a protein loading control. (**D**) Relative band intensity of p16^INK4a^ level. p16^INK4a^ level is well preserved in the IPC+TI group. The bars represent means ± SD (*n* = 7, * *p* < 0.05 versus sham; ^#^ *p* < 0.05 versus TI group).

As shown in Figure 6, TI-induced change and IPC-mediated protection of p16^INK4a^ (endogenous cdk inhibitor) in CA1 pyramidal neurons was investigated. In the sham group, strong p16^INK4a^ immunoreactivity was found in the pyramidal cells (Figure 6A(a)). However, in the TI group, p16^INK4a^ immunoreactivity shown in the CA1 pyramidal neurons was decreased (81.7% at 1 day, 16.3% at 2 days, and 49.3% at 5 days of the sham) (Figure 6A(b–d),B). In the IPC+sham group, p16^INK4a^ immunoreactivity detected in the CA1 pyramidal neurons was similar to that shown in the sham group (Figure 6A(e),B). Additionally, p16^INK4a^ immunoreactivity in the CA1 pyramidal neurons detected in the IPC+TI group did not change after TI (Figure 6A(f–h),B).

#### 3.4.2. p16^INK4a^ Protein Level

As shown in Figure 6C,D, p16I^NK4^^a^ protein level in CA1 with or without IPC was examined after TI. The level of p16I^NK4^ protein was much founded in the sham group (Figure 6C). However, in the TI group, the level of p16I^NK4^ was dramatically reduced (25.6% at 1 day, 15.7% at 2 days, and 16.8% at 5 days of the sham) after TI (Figure 6C,D). In the IPC+sham group, p16I^NK4^ protein level was well preserved in CA1, showing that p16I^NK4^ level was also preserved in the IPC+TI group (Figure 6C,D).

### 3.5. TI Altered p-Rb Expression and IPC Protected the Change in CA1 Pyramidal Cells

#### 3.5.1. *p*-RB Immunoreactivity

**Figure 7:**
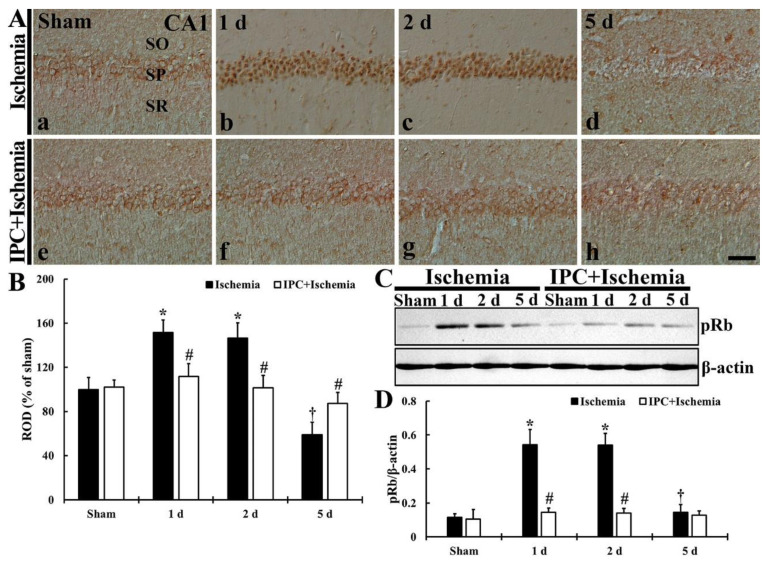
(**A**) Immunostaining of *p*-Rb in CA1 of the TI (upper column) and IPC+TI (lower column) groups at sham (a,e), 1 day (b,f), 2 days (c,g), and 5 days (d,h) after TI. *p*-Rb immunoreactivity is strongly detected in the pyramidal cells at 1 day and 2 days after TI, but very weak at 5 days after TI. However, in the IPC+sham and IPC+TI groups, *p*-Rb immunoreactivity in pyramidal neurons is similar to that shown in the sham group. Scale bar = 50 μm. (**B**) ROD graph of *p*-Rb immunoreactivity in pyramidal cells. ROD was calibrated as percent (sham group, 100%). The bars represent means ± SD (*n* = 7, * p < 0.05 versus sham; *^#^ p* < 0.05 versus TI group; ^†^ *p* < 0.05 versus prior time point of each group). (**C**) Western blot of *p*-Rb (110 kDa) in CA1 of the TI and IPC+TI groups at sham, 1 day, 2 days and 5 days after TI. β-actin is used for a protein loading control. (**D**) Relative band intensity of *p*-Rb level in CA1. The bars represent means ± SD (*n* = 7, * *p* < 0.05 versus sham; ^#^ *p* < 0.05 versus TI group; ^†^ *p* < 0.05 versus prior time point of each group).

In CA1 pyramidal cells, TI-induced alteration and IPC-mediated protection of *p*-Rb immunoreactivity was investigated as shown in Figure 7A,B. In the sham group, *p*-Rb immunoreactivity was weakly found in the cytoplasm of the pyramidal neurons (Figure 7A(a,c)). In the TI group, *p*-Rb immunoreactivity was strongly expressed in the nuclei of the CA1 pyramidal neurons at 1 and 2 days after TI, showing that ROD was 151.7% at 1 day and 146.4% at 2 days when compared with that found in the sham group (Figure 7B). At 5 days post-TI, however, *p*-Rb immunoreactivity detected in the CA1 pyramidal neurons was too weak (59.2% of the sham) at 5 days post-TI (Figure 7A(d),B). In the IPC+sham group, however, *p*-Rb immunoreactivity in the pyramidal cells did not differ from that shown in the sham group (Figure 7A(e),B). Also, *p*-Rb immunoreactivity in the CA1 pyramidal cells of the IPC+TI group was similar to that shown in the IPC+sham group (Figure 7A(f–h),B).

#### 3.5.2. *p*-Rb Protein Level

TI-induced change and IPC-mediated protection of *p*-Rb protein level in CA1 was examined as shown Figure 7C,D. *p*-Rb protein level in the sham group was low (Figure 7C), but *p*-Rb protein level in the TI group was significantly enhanced (472.1% at 1 day and 470.2% and 2 days of the sham) after TI (Figure 7C,D). At 5 days post-TI, *p*-Rb level was reduced (125.1% of the sham) (Figure 7C,D). However, in the IPC+sham and IPC+TI groups, the level of *p*-Rb was similar to that shown in the sham group (Figure 7C,D).

### 3.6. TI Altered E2F1 Expression and IPC Protected the Change in CA1 Pyramidal Cells

#### 3.6.1. E2F1 Immunoreactivity

**Figure 8:**
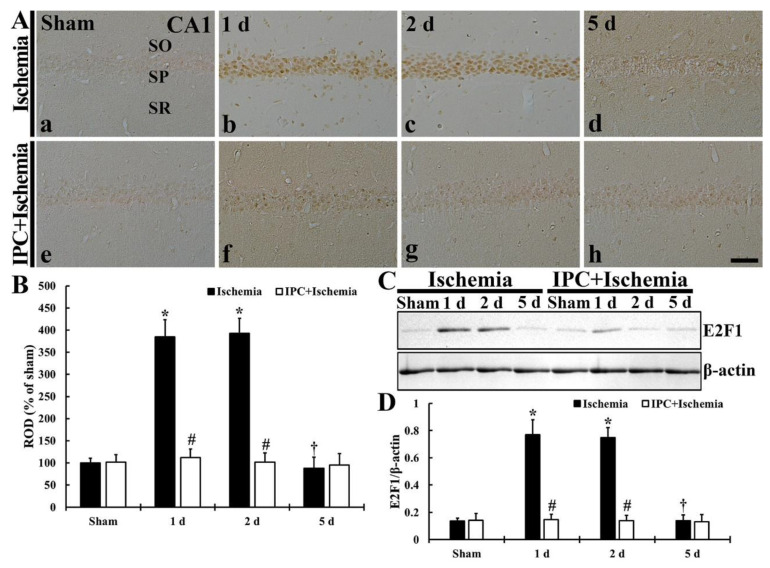
(**A**) Immunostaining of E2F1in CA1 of the TI (upper column) and IPC+TI (lower column) groups at sham (a,e), 1 day (b,f), 2 days (c,g), and 5 days (d,h) after TI. E2F1 immunoreactivity is dramatically increased in the pyramidal cells at 1 day and 2 days after TI, but, in the IPC+TI group, E2F1 immunoreactivity is similar to that shown in the sham group. Scale bar = 50 μm. (**B**) Quantitative graph of E2F1 immunoreactivity in pyramidal cells. ROD was calibrated as percent (sham group, 100%). The bars represent means ± SD (*n* = 7, * p < 0.05 versus sham; *^#^ p* < 0.05 versus TI group; ^†^ *p* < 0.05 versus prior time point of each group). (**C**) Western blot of E2F1 (60 kDa) in CA1 of the TI and IPC+TI groups at sham, 1 day, 2 days and 5 days after TI. β-actin is used for a protein loading control. (**D**) Relative band intensity of E2F1 level. The bars represent means ± SD (*n* = 7, * *p* < 0.05 versus sham; ^#^ *p* < 0.05 versus TI group; ^†^ *p* < 0.05 versus prior time point of each group).

As shown in Figure 8A,B, TI-induced change and IPC-mediated protection of E2F1 immunoreactivity of CA1 pyramidal cells was studied. In the sham group, E2F1 immunoreactivity in the pyramidal neurons was too weak (Figure 8A(a)). In the TI group, however, E2F1immunoreactivity in the CA1 pyramidal cells was markedly increased (385.0% at 1 day and 392.6% at 2 days of the sham) after TI (Figure 8A(b,c),B). However, at 5 days post-TI, E2F1immunoreactivity in the CA1 pyramidal neurons was too weak (87.7% of the sham) (Figure 8A(d),B). In the IPC+sham group, E2F1 immunoreactivity in the CA1 pyramidal neurons did not differ from that found in the sham group (Figure 8A(e),B). E2F1 immunoreactivity in the pyramidal neurons, in the IPC+TI group, was slightly increased at 1 day and 2 days after TI, however, E2F1 immunoreactivity in the CA1 pyramidal cells was too weak at 5 days post-TI (Figure 8A(f–h),B).

#### 3.6.2. E2F1 Protein Level

As shown in Figure 8C,D, TI-induced change and IPC-mediated protection of E2F1 protein level in CA1 was analyzed. The level of E2F1 protein in the sham group was low (Figure 8C). However, in the TI group, E2F1 protein level was enhanced (568.7% at 1 day and 553.5% at 2 days of the sham) after TI (Figure 8C,D). Five days after TI, however, the level of E2F1 was reduced to the sham level (Figure 8C,D). In the IPC+sham and IPC+TI groups, E2F1 protein level was not different form that shown in the sham group (Figure 8C,D).

## 4. Discussion

Brain (cerebral) ischemia happens when blood flow in the brain is reduced to a level that causes the deprivation of oxygen and glucose, and can lead to a pathological state, including neuronal damage/loss and inflammation [41,42]. Gerbils have been used for transient forebrain (telencephalon) ischemia by ligation of both (left and right) common carotid arteries to study the mechanisms of selectively delayed neuronal cell death following the ischemia, because the gerbils do not have the posterior communicating arteries, which connect the internal carotid to vertebral arteries in Willis’ circle [43]. Especially, the pyramidal neurons (cells), as principal cells in CA1 of the hippocampus, die at a few (4–5) days after transient ischemia [5,44]. Until now, the exact mechanisms underlying the delayed death of the CA1 pyramidal cells after transient ischemia have not fully been elucidated yet.

It is well permitted that IPC is able to induce tolerance in neurons to a subsequent longer (lethal) transient ischemia in animal models. [8,9,10,11,12,45]. IPC time period has been determined in gerbils [17]. Namely, at least a 1-day interval between IPC (2-min ischemia) and subsequent morbid (5-min) ischemia in the hippocampus is necessary to induct the protection of the CA1 pyramidal cells from the 5-min ischemia. In our current study, we also obtained the results showing that IPC (2-min transient forebrain ischemia) protected the CA1 pyramidal cells from 5 min of transient ischemia developed in gerbil forebrain. At five days after the morbid ischemia, pyramidal neurons located in CA1 showed a typical feature of neuronal cell death in CV histochemistry, immunostaining with NeuN and histofluorescence with F-J B, but the pyramidal cells were significantly protected from the morbid ischemia by IPC. Although, to date, IPC has provided significant protection against ischemic cerebral injury, we should understand its mechanisms to improve therapeutic strategies for ischemic brain injuries.

Cyclin D1 has been studied in many in vivo (animal) models of neuronal cell death and/or degeneration following brain diseases such as brain ischemia [28,29,46,47]. In a rat model of transient focal brain ischemia, elevated cyclin D1 is predominantly detected in morphologically intact or damaged neurons that are localized to ischemic core in early time after the ischemia, suggesting that cyclin D1 may play an important role in promoting neuronal survival rather than damage/death [28,42]. In contrast, other researches demonstrate that cyclin D1 mRNA and/or protein are upregulated in astrocytes and microglia located in the hippocampal CA1, rather than in pyramidal neurons, in rat models of transient forebrain or transient global brain ischemia [48,49,50]. However, growing evidence has suggested that cyclin D1 may participate in a signal for death rather than cell division in postmitotic neurons. For instance, in a rat model of transient global cerebral ischemia, cyclin D1 expression in the hippocampus peaks in CA1 pyramidal neurons before nuclear condensation and the appearance of DNA fragmentation, which suggests that cyclin D1 acts as an apoptosis modulator when it is expressed at high level in damaged neurons [29]. In mouse brains with transient focal ischemia, cyclin D1 protein is present in neurons within the infarcted region after ischemic insult, suggesting that its presence is associated with DNA fragmentation and is involved in neuronal death process [41]. In gerbil forebrain with transient ischemia, cyclin D1 is upregulated in pyramidal neurons located in CA1 of the hippocampus following ischemia-reperfusion insult [47]. In addition, in a rabbit model of transient ischemia in the spinal cord, cyclin D1 protein is upregulated in motor neurons, which die eventually, in the early time after ischemic insult, indicating that cyclin D1 is implicated in the process of programmed cell death after transient spinal cord ischemia [29,46,51]. In particular, in ischemic human brain, cyclin D1 is upregulated in nuclei and cytoplasm of scattered neurons within infarcted tissue after cardiac arrest [52]. Like the above-mentioned studies, in our current experiment, we found that cyclin D1 was significantly enhanced in the CA1 pyramidal neurons in response to a neuronal death at 1 day and 2 days after TI, and the increased cyclin D1 expression following TI was controlled by IPC. On the basis of our and previous results, we strongly suggest that IPC could downregulate cyclin D1 to prevent the process of neuronal death following ischemic insults.

It was reported that cyclin D1 increase is accompanied by increases in cdk4 and cyclin D1/cdk4 complex to translocate to the nucleus in a rat model of focal brain ischemia [28]. Based on this finding, we studied the expression of cdk4 in the CA1 pyramidal cells after TI and found that cdk4 immunoreactivity was detected only in the cytoplasm of the intact CA1 pyramidal neurons, but cdk4 was strongly expressed in their nuclei at 1 day and 2 days after TI. This finding means that the increased cdk4 was controlled by IPC. For this finding, Rashidian et al. (2005) reported that, in ischemia/hypoxia in vitro and in vivo, a reduction of cdk4 expression in ischemic neurons was protective against neuronal apoptosis following ischemia/hypoxia [53]. Taken together, we suggest that cdk4 signal plays important roles in transducing neuronal death signal following ischemic insults.

Another cause for increased cdk4 activity could be post-ischemic destruction of cyclin-dependent kinase inhibitors (CDKIs) [54]. Among all CDKIs, p16^INK4a^ specifically induces cell cycle arrest by inhibiting cdk4 activity during brain development [55,56]. A paper using a mouse model of transient focal brain ischemia by Katchanov et al. (2001) shows that p16^INK4a^ is downregulated most neurons after ischemia-reperfusion, and p16^INK4a^-negative neurons are positive to TUNEL and undergo the disintegration of cytoskeleton [49]. Therefore, the authors suggest that p16^INK4a^ is a survival factor and its early downregulation predicts neuronal death after the ischemia. Based on this paper, we propose that the loss of p16^INK4a^ in neurons following an ischemia insult might be paralleled by cyclin D1 expression with a close cdk4 activation, which might be crucial for neuronal death after the ischemic insult. Eventually, we found strong p16^INK4a^ immunoreactivity in intact CA1 pyramidal cells, and that p16^INK4a^ was profoundly downregulated at 2 days after TI, but IPC inhibited the TI-induced downregulation of p16^INK4a^ expression in the neurons. Therefore, we suggest that p16^INK4a^ may be a survival factor in neuronal cells under an ischemic condition.

Previous data suggest that the phosphorylation of Rb protein is mediated via cyclin D1/cdk4 pathway [57]. Accumulating evidence has confirmed that *p*-Rb is highly related to neuronal death under ischemia in vitro and in vivo [58,59]. Importantly, it has been reported that the expression of mutant *p*-Rb prevents neuronal cell death following DNA damage and/or hypoxia [53,60]. In addition, it has been demonstrated that the phosphorylation of Rb by the cyclin D1/cdk4 complex leads to the dissociation of *p*-Rb from its binding partner E2F1 [61]. These needed to investigate whether cyclin D1/cdk4 might be relevant to the phosphorylation of Rb protein after ischemic insults. In our current study, we examined whether Rb acted as a downstream mediator of cdk4 after TI in gerbil brains by using immunohistochemistry and quantitative immunoblotting with a phosphor-epitope-specific antibody and found that *p*-Rb expression following TI was significantly increased in the CA1 pyramidal cells at 1 day and 2 days after TI, which was controlled by IPC. In line with the finding of change in *p*-Rb expression in the CA1 pyramidal cells, we found that E2F1 expression was also significantly enhanced in the CA pyramidal cells at 1 day and 2 days after TI and that IPC controlled the expressions of E2F1 in the neurons after TI, and we suggest that, during the phosphorylation of Rb, the loss of the regulatory binding of Rb is deeply related to the enhancement of E2F1 expression in ischemic neurons. For this finding, a paper by Jin et al. (2001) shows that mRNA encoding the transcription factor E2F1 increases in the hippocampus of rats subjected to 15-min global brain ischemia [58]. In addition, the absence of E2F1 attenuates ischemic damage in mouse models of focal brain ischemia [62,63,64]. Taken together, it is suggested that cdk4 transduces the delayed ischemic death signal, at least through the phosphorylation of Rb. In addition, cell cycle reactivation in neurons following ischemic insult is a sign of death through the dissociation of E2F1/Rb complex and E2F1 release.

Mechanisms underlying IPC-induced neuroprotection have been diversely suggested. For some instances, IPC increases small ubiquitin-like modifier (SUMO) conjugation (also called SUMOylation), which is regarded as conferring tolerance against an ischemic insult [65]. It has been also known that SUMOylation due to IPC targets Na^+^/Ca^2+^ exchangers (NCXs), which play a neuroprotective role by attenuation of ischemia-induced excitotoxicity [65,66,67]. In addition, we previously demonstrated that IPC-mediated neuroprotection was accomplished by upregulation of hypoxia-inducible factor-1α (HIF-1α), which enhanced vascular endothelial growth factor (VEGF) expression and nuclear factor-κB (NF-κB) activation in CA1 pyramidal neurons [68].

## 5. Conclusions

Our current findings showed that IPC apparently protected CA1 pyramidal cells from ischemic injury induced by a subsequent real TI. This IPC-mediated neuroprotection was significantly associated with the downregulation of cyclin D1 and cdk4. In addition, the downregulation of *p*-Rb and the upregulation of E2F1 by IPC are closely implicated with the protection of the CA1 pyramidal neurons from the ischemic injury. Furthermore, the maintenance of p16^INK4a^ by IPC was implicated with the protection of cyclin D1/cdk4-dependent neuronal death following TI. Taken together, although our present results may be insufficient in explaining the mechanisms of IPC in transient forebrain ischemia, we strongly suggest that the downregulation of cyclin D1/cdk4 and the maintenance of p16^INK4a^ is critical in neuroprotection against ischemic injury induced by TI.

## Data Availability

The data presented in this study are available on request from the corresponding author.

## Abbreviations

CA1	cornu ammonis 1
CV	cresyl violet
Cdk4	cyclin-dependent kinase 4
E2F1	E2 promoter binding factor 1
F-JB	fluoro-jade B
GFAP	glial fibrillary acidic protein
Iba-1	ionized calcium-binding adapter molecule 1
IPC	ischemic preconditioning
NeuN	neuronal nuclear antigen
Rb	retinoblastoma
OD	optical density
ROD	relative optical density
SO	stratum oriens
SP	stratum pyramidale
SR	stratum radiatum
TI	transient ischemia.
